# The HOMA-Adiponectin (HOMA-AD) Closely Mirrors the HOMA-IR Index in the Screening of Insulin Resistance in the Brazilian Metabolic Syndrome Study (BRAMS)

**DOI:** 10.1371/journal.pone.0158751

**Published:** 2016-08-04

**Authors:** Brunna Sullara Vilela, Ana Carolina Junqueira Vasques, Roberta Soares Lara Cassani, Adriana Costa e Forti, José Carlos Pareja, Marcos Antonio Tambascia, BRAMS Investigators, Bruno Geloneze

**Affiliations:** 1 LIMED - Laboratory of Investigation on Metabolism and Diabetes, Gastrocentro, University of Campinas, Campinas, Sao Paulo, Brazil; 2 School of Applied Sciences, University of Campinas, Limeira, Sao Paulo, Brazil; 3 Department of Endocrinology, Federal University of Ceara, Fortaleza, Brazil; 4 Department of Endocrinology, University of Campinas, Sao Paulo, Brazil; Weill Cornell Medical College Qatar, QATAR

## Abstract

**Background:**

The major adverse consequences of obesity are associated with the development of insulin resistance (IR) and adiposopathy. The Homeostasis Model Assessment-Adiponectin (HOMA-AD) was proposed as a modified version of the HOMA1-IR, which incorporates adiponectin in the denominator of the index.

**Objectives:**

To evaluate the performance of the HOMA-AD index compared with the HOMA1-IR index as a surrogate marker of IR in women, and to establish the cutoff value of the HOMA-AD.

**Subjects/Methods:**

The Brazilian Metabolic Syndrome Study (BRAMS) is a cross-sectional multicenter survey. The data from 1,061 subjects met the desired criteria: 18–65 years old, BMI: 18.5–49.9 Kg/m² and without diabetes. The IR was assessed by the indexes HOMA1-IR and HOMA-AD (total sample) and by the hyperglycemic clamp (n = 49). Metabolic syndrome was defined using the IDF criteria.

**Results:**

For the IR assessed by the clamp, the HOMA-AD demonstrated a stronger coefficient of correlation (r = -0.64) compared with the HOMA1-IR (r = -0.56); p < 0.0001. In the ROC analysis, compared with the HOMA1-IR, the HOMA-AD showed higher values of the AUC for the identification of IR based on the clamp test (AUC: 0.844 vs. AUC: 0.804) and on the metabolic syndrome (AUC: 0.703 vs. AUC: 0.689), respectively; p < 0.001 for all. However, the pairwise comparison did not show evidence of superiority for the HOMA-AD in comparison with the HOMA1-IR in the diagnosis of IR and metabolic syndrome (p > 0.05). The optimal cutoff identified for the HOMA-AD for the diagnosis of IR was 0.95.

**Conclusions:**

The HOMA-AD index was demonstrated to be a useful surrogate marker for detecting IR among adult women and presented a similar performance compared with the HOMA1-IR index. These results may assist physicians and researchers in determining which method to use to evaluate IR in light of the available facilities.

## Introduction

The major adverse health consequences of obesity are especially associated with the development of insulin resistance (IR) and adiposopathy. IR is clinically defined as a failure of a known quantity of exogenous or endogenous insulin to increase glucose uptake and the consumption of target organs in an individual as much as it does in a healthy population. The spectrum of metabolic disorders associated with IR extends further than type 2 diabetes (T2DM) and includes dyslipidemia, hypertension, hypercoagulability and inflammation, all of which are related to the metabolic syndrome and are risk factors for cardiovascular disease [1].

Adiposopathy is characterized by the deposition of ectopic adipose tissue with dysfunctional metabolic properties [2]. Adiponectin is secreted by the adipose tissue and plays an increasingly important role in IR. The adiponectin levels are reduced in obese individuals, are significantly restored to normal levels after weight loss, and are negatively correlated with IR. In addition, low plasma adiponectin was demonstrated to be an independent risk factor for the development of T2DM [3]. The link between adiponectin and IR is related to the ability of adiponectin to mediate an insulin-sensitizing effect by binding to its receptors, AdipoR1 and AdipoR2, resulting in the activation of AMPK, PPAR-α, and other possible, yet-unknown signaling pathways [4].

The assessment of IR in clinical practice and in epidemiological studies is of great importance. The Homeostasis Model Assessment-Insulin Resistance (HOMA1-IR) is widely used and has been validated against the clamp technique, which is the gold standard method to assess IR. The HOMA1-IR index is based on the fasting glucose and insulin levels, and no distinction is made between hepatic and peripheral IR [5]. More recently, a study conducted in the general Japanese population proposed the Homeostasis Model Assessment–Adiponectin (HOMA-AD) index, a modified version of the model, which incorporates the total serum adiponectin level in the denominator of the index and, consequently, it adds an indirect measurement of adiposopathy [6], and ultimately an adjustment to the individual degree of adiposity. Subsequently, studies with obese children [7] and with chronic hemodialysis patients [8] have also identified the good performance of the HOMA-AD index, whereas one study with lean non cirrhotic HCV outpatients did not detect any benefit when comparing the HOMA-AD with the HOMA1-IR [9].

Therefore, the present study aimed 1) to investigate the correlations between the HOMA-AD index with clinical, anthropometric and metabolic parameters related to IR; 2) to evaluate the performance of the HOMA-AD index compared to the HOMA1-IR index as a useful surrogate marker of IR, as assessed using a hyperglycemic clamp test in women with a wide range of adiposity; and 3) to establish the cutoff value of the HOMA-AD for screening IR.

## Methods

### Subjects

The present study comprised a cross-sectional analysis from the Brazilian Metabolic Syndrome Study (BRAMS), a multicenter survey, which included individuals from three different States in Brazil: São Paulo, Ceara and Minas Gerais. A total of 5,668 subjects were assessed. The subjects who were invited to participate were selected from outpatient clinics for the metabolic syndrome and obesity or through local and internet advertisements.

The samples were selected using an intentional non-probabilistic sampling. The data from the 1,061 subjects met the following desired criteria: female sex, aged from 18 to 65 years, BMI from 18.5 to 49.9 kg/m^2^, and without diabetes according to the ADA recommendations [10]. Afterward, the healthy group (n = 550) was defined as: BMI < 30 kg/m², HDL cholesterol ≥ 40 mg/dL, triglycerides ≤ 200 mg/dL, LDL cholesterol < 160 mg/dL, fasting plasma glucose < 100 mg/dL and normotensive status [11]. None of the subjects were taking medications that affected glycemia or insulin sensitivity. The exclusion criteria were as follows: clinical or laboratory evidence of cardiac, renal, liver or endocrine disease; severe systemic disease (e.g., cancer, heart failure and AIDS), heavy alcohol consumers (five or more units on each of five or more days in the past 30 days), as well as women who were body builders, athletes, pregnant or lactating. The presence of polycystic ovary syndrome was not included in the study exclusion criteria.

The study was approved by the Ethics Committee of the University of Campinas, Brazil. All of the participants signed informed consent before participation.

### Anthropometrical and body composition assessment

All of the examiners were trained by one registered dietitian to perform the anthropometric measurements using standard techniques. The subjects underwent an anthropometric examination without heavy clothing and shoes. Height was determined using a stadiometer with a length of 220 cm and subdivided into 0.1 cm segments. Weight was measured on an electronic digital scale positioned on a flat surface, with a maximum capacity of 200 kg and a sensitivity of 100 g. Body Mass Index (BMI) was calculated as weight (in kilograms) divided by the square of height (in meters). Waist circumference was measured in the standing position by a flexible and inelastic measuring tape (TBW Ltda, São Paulo, Brazil) at the umbilicus level after a normal exhalation without clothing in the measurement area and taking the necessary care not to compress the tissues. The amount of fat-free mass was determined using a bioimpedance analyzer—model BIA 310 (lean body mass SEE = 1.4 kg, r = 0.97), according to the manufacturer’s protocol (Biodynamics Corporation, Seattle, USA).

### Insulin resistance assessment

IR was assessed by surrogate indexes obtained in the fasting state and by a dynamic hyperglycemic clamp test.

The surrogate indexes of IR comprised the HOMA1-IR and HOMA-AD indexes. The HOMA1-IR was calculated as follows: [fasting glucose (mmol/L) x fasting insulin (mU/L)] / 22.5 [5].According to a previous cutoff determined for the non-diabetic adult Brazilian population, the HOMA1-IR values above 2.71 were considered for IR. The study population of this study overlaps the population used to establish the cutoff point for the Brazilian population, in which there was no difference according to gender regarding the distribution of the HOMA1-IR index [12]. In addition, considering that the Brazilian population is a mixed population and do not has different ethnic groups, a single cut point was determined in the initial publication. Despite the fact that, HOMA2-IR theoretically incorporates physiological adjustments accounting for variations in hepatic and peripheral glucose resistance, and renal glucose losses, thus, providing a more accurate IR index; in the context of the present study the HOMA1-IR index was applied due to its wide application in published research studies validating their data against the clamp method [11, 13].

The HOMA-AD was calculated with the formula: [fasting glucose (mmol/L) x fasting insulin (mU/L)] / [22.5 x fasting adiponectin (μg/ml)] [8].

The hyperglycemic clamp test provides an accurate measurement of IR. A previous study compared the insulin sensitivity indices (ISI) that were obtained during euglycemic and hyperglycemic clamp tests and demonstrated that both indices yield comparable IR estimates, with a high coefficient of correlation (r = 0.84; p < 0.0001) [14]. In the present study, the hyperglycemic clamp test was applied as the reference method for validating the HOMA-AD index as a surrogate marker of IR.

A random subsample of 49 subjects (5% of the total sample) underwent the hyperglycemic clamp test. All of the tests were performed at 8 a.m. and after a 12-h overnight fast. The blood glucose levels were raised to the desired plateau (180 mg/dl) for three hours, following a previously detailed protocol [15]. IR was expressed by the insulin sensitivity index (ISI) that was calculated with the following formula: ISI = [average glucose infusion rate adjusted for free fat mass during the last hour of the test / average plasma insulin levels during the last hour of the test]. The subjects who were below or equal to the first tertile (0.18 mg.kg^-1^.mim^-1^.μU/l*100) of the ISI were considered to be insulin resistant.

### Definition of metabolic syndrome

Metabolic syndrome was defined using the International Diabetes Federation criteria [16], which includes central obesity based on waist circumference ≥ 80 cm for women, plus any two of the following factors: raised triglyceride levels ≥ 150 mg/dl or current treatment for this condition; reduced HDL cholesterol < 50 mg/dl in women or current treatment or this lipid; raised blood pressure (systolic blood pressure ≥ 130 or diastolic blood pressure ≥ 85 mmHg) or treatment of previously diagnosed hypertension; or raised fasting plasma glucose ≥ 100 mg/dl.

### Assays

The blood samples were obtained after a 12-h overnight fast and were stored at -20°C for later evaluation. The total cholesterol (K 083, Bioclin), high-density lipoprotein cholesterol (K 015, Bioclin), triglycerides (K 117, Bioclin), and gamma-glutamyltransferase (K 080, Bioclin) were measured using automated enzymatic and colorimetric methods. The LDL-cholesterol levels were calculated with the Friedewald equation [17]. The ultra-sensitivity C-reactive protein was determined using a nephelometric assay with a sensitivity of 0.01mg/dl. The plasma glucose levels were promptly measured in the fasting state and during the clamp tests using a glucose analyzer (YSI 2700; YSI Life Sciences, Yellow Spring, OH, USA) with a CV of 2%. The plasma insulin levels were analyzed using an automated two-site chemiluminescent immunometric assay (Immulite 1000 System; Siemens Health Diagnostics, USA). The intra- and inter-assay CVs were 5.2–6.4% and 5.9–8.0%, respectively, for insulin. The adiponectin levels were measured using an ELISA (Quantikine Human Total Adiponectin Immunoassay; Linco Research), the intra- and inter-assay CVs were 2.5–4.7% and 5.8–6.9%, respectively, and the coefficients of variation were below 10%.

### Statistical analysis

Statistical analyses were performed using IBM SPSS-Statistics version 20.0. The Mann Whitney test was applied to compare the total sample with the subsample, and the data were expressed as the median and interquartile range. The analysis of covariance (ANCOVA) followed by the Bonferroni post hoc test was calculated considering age and BMI as covariates and was applied to compare the distribution of the clinical, anthropometric and metabolic variables according to the normal groups established for the HOMA1-IR index (< 2.71) and adiponectin levels (> 1^st^ quartile). The Spearman’s correlation coefficient was applied for binary correlations. The receiver operation characteristic (ROC) curves for the HOMA-AD and HOMA1-IR indices were built to assess the performance of both in identifying IR according to the hyperglycemic clamp (subsample) and the IDF criteria for the metabolic syndrome (total sample). The method of Hanley & McNeil [18] was applied to test the statistical significance of the difference between the areas under the ROC curve (AUC). The cut-off value for IR was based on the 90th percentile in the healthy group. Significance was set at p < 0.05.

## Results

The clinical, anthropometric and metabolic characteristics of the participants are summarized in Table 1. In general, none of the variables differed between the groups, except for the gamma-glutamyltransferase, fasting glucose and fasting insulin levels, though the values were in the non-diabetic range for both of the groups. Hence, the great majority of patients (86%) had fasting glucose below 100 mg/dL.

**Table 1:** Clinical, anthropometric and metabolic characteristics of the study participants.

Variables	Total samplen = 1061	Clamp samplen = 49	P-value
**Age (years)**	35 (25–47)	40 (29–47)	0.056
**Body mass index (kg/m²)**	26.8 (23.4–31.4)	26.7 (23.1–32.5)	0.821
**Waist circumference (cm)**	90.5 (80.5–100.6)	92.0 (81.7–104.0)	0.344
**Systolic blood pressure (mmHg)**	117 (110–122)	112 (105–120)	0.584
**Diastolic blood pressure (mmHg)**	80 (70–80)	80 (70–88)	0.350
**Triglycerides (mg/dl)**	94 (69–129)	95 (57–123)	0.401
**Total cholesterol (mg/dl)**	184 (158–210)	190 (164–202)	0.863
**LDL cholesterol (mg/dl)**	110 (89–131)	109 (86–124)	0.575
**HDL cholesterol (mg/dl)**	50 (43–60)	53 (46–63)	0.111
Fasting Glucose (mg/dl) *	82 (76–90)	89 (81–96)	0.001
Fasting Insulin (mU/l) *	8.5 (5.7–13.0)	7.0 (4.3–12.1)	0.043
**HOMA1-IR**	1.70 (1.1–2.7)	1.55 (0.94–2.65)	0.176
**Adiponectin (μg/ml)**	3.57 (2.43–5.57)	3.30 (1.97–5.10)	0.078
**HOMA-AD**	0.47 (0.24–0.89)	0.48 (0.24–0.98)	0.882
Gamma-glutamyltransferase (U/l)*	19.0 (14.0–27.0)	15.0 (12.0–22.0)	0.015
**C-reactive protein (mg/dl)**	0.27 (0.13–0.69)	0.27 (0.08–0.46)	0.208

The data are presented as the median (percentiles 25–75).

To explore the hypothesized favorable combination of the adiponectin levels with the HOMA1-IR index in the identification of the clinical and metabolic alterations related to the IR, the normal and altered groups for both of the desired parameters were put together simultaneously in four possible combinations (Table 2).

**Table 2:** Comparisons between the distribution of the clinical, anthropometric and metabolic variables adjusted by age and BMI according to the normal groups established for the HOMA1-IR index and adiponectin levels.

Variables	Group 1 n = 635	Group 2 n = 162	Group 3 n = 162	Group 4 n = 102	P-value
Age (years)*	36 ± 1	38 ± 1	37 ± 1	38 ± 1	0.109
BMI (kg/m²)*	26.2 ± 0.2	28.9 ± 0.5^a^	30.6 ± 0.5^a^^b^	34.9 ± 0.7 ^a^^b^^c^	0.001
**Waist circumference (cm)**	90.8 ±0.3	92.2 ± 0.6	91.6 ± 0.6	92.8 ± 0.8^a^	0.042
**Systolic blood pressure (mmHg)**	116.4 ± 0.6	117.2 ± 1.1	117.4 ± 1.1	119.8 ± 1.5	0.225
**Diastolic blood pressure (mmHg)**	76.0 ± 0.4	76.5 ± 0.8	76.6 ± 0.8	76.9 ± 1.1	0.872
**Triglycerides (mg/dl)**	98.4 ± 2.3	105.1 ± 4.4	124.7 ± 4.6 ^a^^b^	143.3 ± 6.0 ^a^^b^	0.001
**Total cholesterol (mg/dl)**	182.1 ± 1.6	186.1 ± 3.0	194.4 ± 3.1 ^a^	195.3 ± 4.0 ^a^	0.001
**HDL cholesterol (mg/dl)**	53.0 ± 0.5	51.5 ± 1.0	52.6 ± 1.1	49.0 ± 1.4	0.057
**Gamma-glutamyltransferase (U/l)**	22.1 ± 1.1	24.2 ± 2.0	29.8 ± 2.2 ^a^	37.3 ± 2.6 ^a^^b^	0.001
**C-reactive protein (mg/dl)**	0.83 ± 0.16	0.59 ± 0.31	1.40 ± 0.28	1.59 ± 0.37	0.074

Group 1: Normal HOMA1-IR (HOMA1-IR index value < 2.71) and adiponectin levels (adiponectin level > 1st quartile); Group 2: Normal HOMA1-IR (HOMA1-IR index value < 2.71) and decreased adiponectin levels (adiponectin level < 1st quartile); Group 3: Increased HOMA1-IR (HOMA1-IR index value > 2.71) and normal adiponectin levels (adiponectin level > 1st quartile); Group 4: Increased HOMA1-IR (HOMA1-IR index value > 2.71) and decreased adiponectin levels (adiponectin level < 1st quartile).

The data are expressed as the mean ± standard error of the mean adjusted by age and BMI. ANCOVA followed by the Bonferroni post hoc test.

* Not adjusted by age and BMI

^a^ p < 0.05 vs Group 1

^b^ p < 0.05 vs Group 2

^c^ p < 0.05 vs Group 3

The only difference found between Groups 1 and 2 was the presence of higher BMI levels in Group 2 (p < 0.05). Group 3 had higher levels of BMI and triglycerides compared to Groups 1 and 2 (p < 0.05). Moreover, Group 3 had higher total cholesterol and gamma-glutamyltransferase levels than the Group 1 (p < 0.05). Hence, the exclusive presence of increased HOMA-IR was mainly related to an undesirable cardiometabolic and adiposity profile than the exclusive presence of decreased adiponectin. Finally, the Group 4 showed the same performance as Group 3 in comparison to Groups 1 and 2; except for BMI levels which were increased in Group 4 in comparison to the others (p < 0.05). These results demonstrate that after adjustment for age and BMI, the presence of altered adiponectin did not result in worse values for the studied parameters.

**Fig 1:**
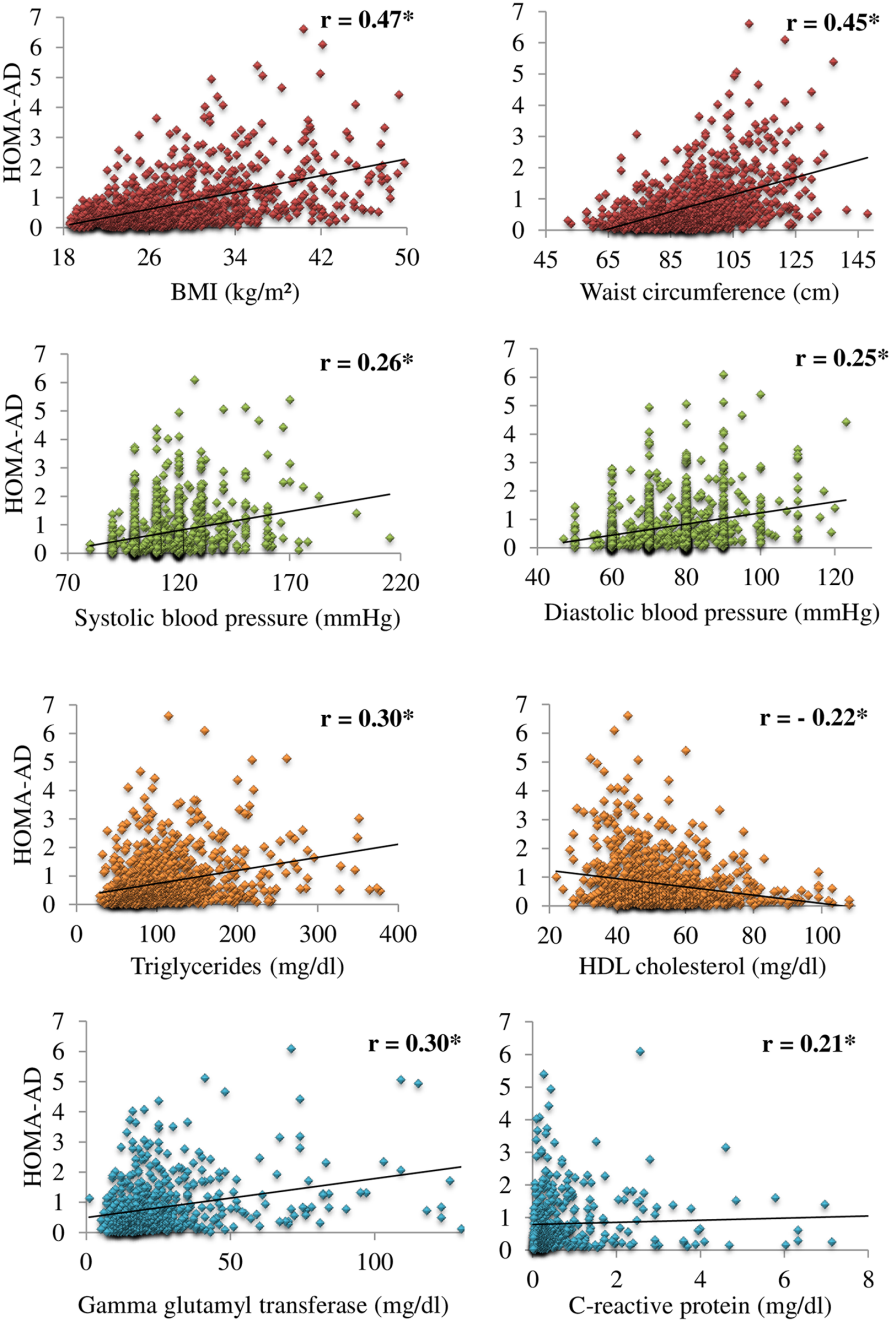
Scatter plots demonstrating the bivariate correlations between the HOMA-AD index with the clinical and metabolic components related to the metabolic syndrome. *p < 0.001.

**Fig 2:**
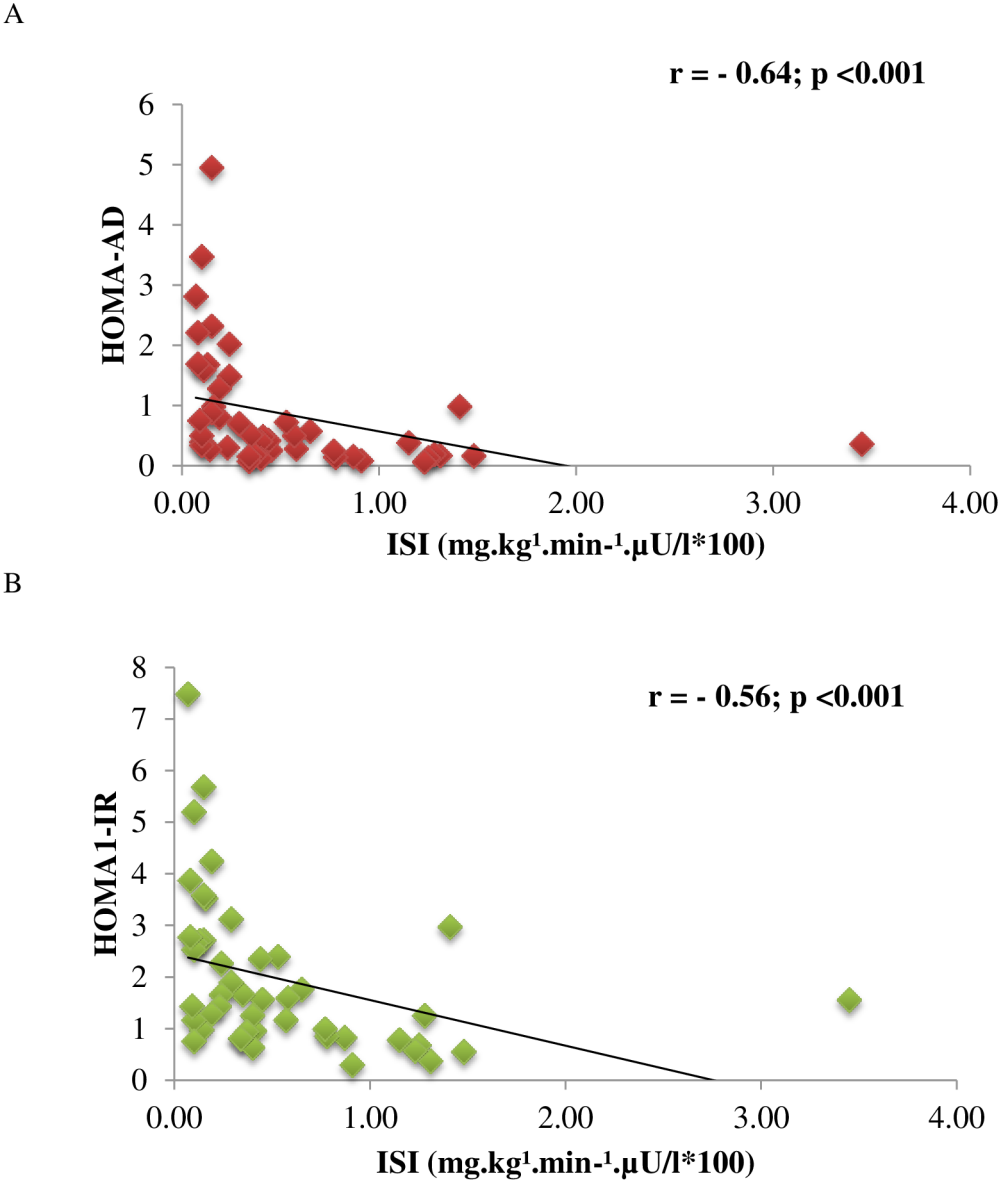
Bivariate correlation coefficients between the surrogate markers of insulin resistance, HOMA-AD (A) and HOMA1-IR (B), with the insulin sensitivity index obtained in the hyperglycemic clamp test. Subsample n = 49.

The bivariate correlations between the HOMA-AD index with the clinical and metabolic components related to the metabolic syndrome are shown in Fig 1. Highly significant coefficients of correlation were observed, ranging from weak to moderate (p<0.001). For the IR (Fig 2), the HOMA-AD index demonstrated a stronger coefficient of correlation with the insulin sensitivity index obtained in the hyperglycemic clamp test compared with the HOMA1-IR index.

**Fig 3:**
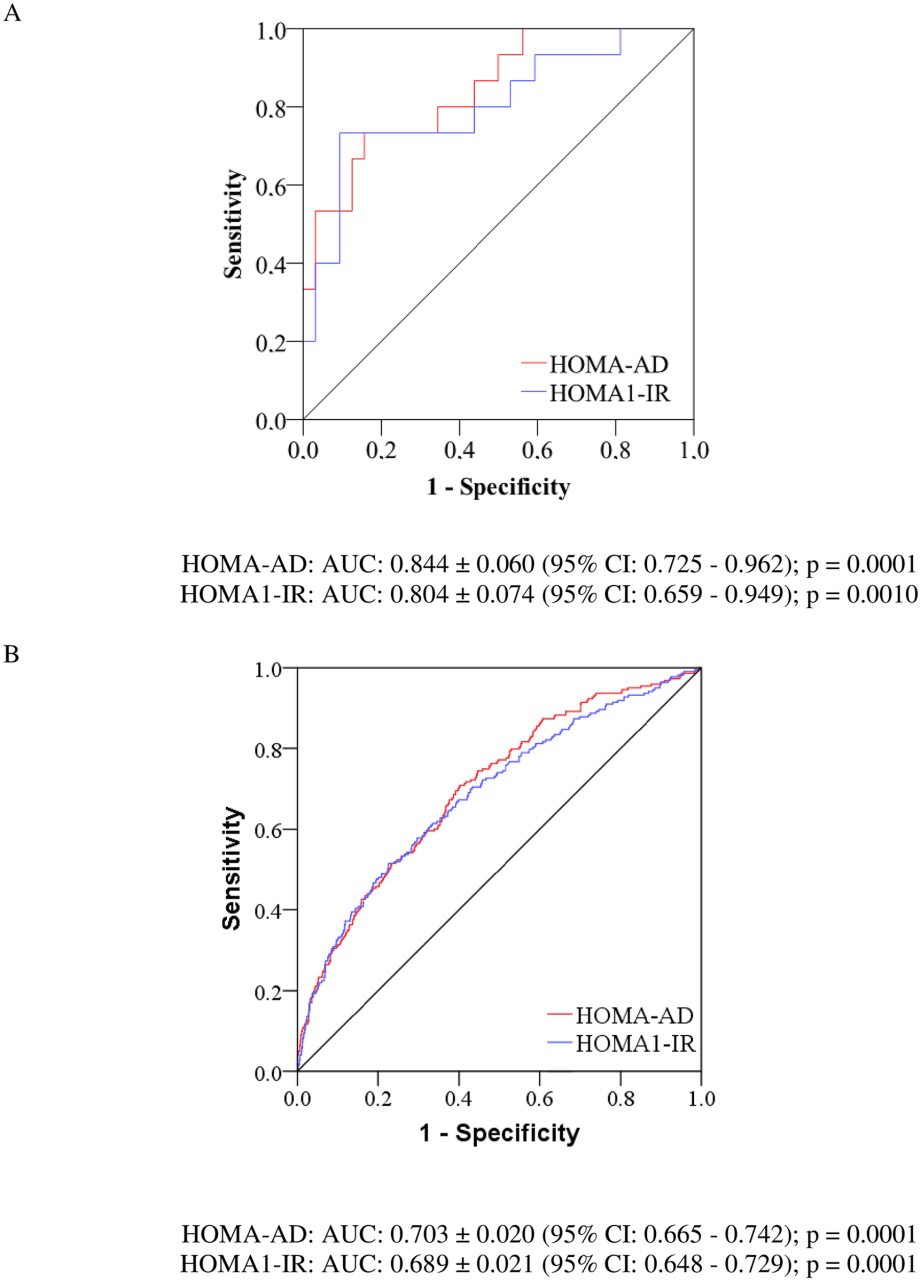
Receiver operating characteristic curves for the HOMA-AD and HOMA1-IR indices in identifying insulin resistance by the hyperglycemic clamp and metabolic syndrome in the total sample.

In the ROC analysis (Fig 3), the values for the AUC above 0.70 are generally considered to show good discriminatory capacity. The HOMA-AD index compared with the HOMA1-IR showed higher values of the AUC for the identification of IR based on the clamp test and on the metabolic syndrome criteria. However, the pairwise comparison did not show evidence of superiority for the HOMA-AD index in comparison with the HOMA1-IR index in either considering the diagnosis of IR in the clamp test (Z statistic = 1.022; p = 0.307) or considering the metabolic syndrome criteria (Z statistic = 0.956; p = 0.339).

Finally, the optimal cut-off value for the HOMA-AD index in the screening of insulin resistance was 0.95, considering the healthy subjects whose HOMA1-IR values were above the 90th percentile.

## Discussion

IR is a condition in which the normal insulin level fails to promote glucose homeostasis, favoring the development of metabolic and cardiovascular diseases [1]. Among a diversity of IR markers that exhibit varying degrees of accessibility and accuracy, the HOMA-AD, which is an adipokine-based IR index, has been proposed as a novel, simple and accurate surrogate marker of IR [6–8]. In this context, the present study aimed to evaluate the performance of the HOMA-AD index compared with the well-established HOMA1-IR index in Brazilian women with a wide range of adiposity.

The main findings were as follows: 1) the HOMA-AD significantly correlated to the clinical, anthropometric and metabolic parameters related to IR; 2) the HOMA-AD was demonstrated to be a useful surrogate marker of IR and metabolic syndrome; 3) the HOMA-AD demonstrated similar performance as the HOMA1-IR for identifying IR; and 4) the optimal cutoff identified for the HOMA-AD index for identifying IR in adult women was 0.95.

Until now, very few studies had investigated the performance of the HOMA-AD index in the assessment of IR. One such study was conducted with Japanese subjects of both sexes with various degrees of glucose tolerance [6]. The authors identified that IR assessed by the euglycemic hyperinsulinemic clamp technique was more significantly correlated with the HOMA-AD index (r = -0.64) than the HOMA1-IR index (r = -0.59); p < 0.001 for all. In the present study, the values obtained for the hyperglycemic clamp were close to those mentioned earlier: HOMA-AD (r = -0.64; p < 0.0001) and HOMA1-IR (r = -0.56; p < 0.0001). Another observational study involving a very small sample of adult patients on chronic hemodialysis demonstrated that the HOMA-AD index (r = -0.67, p < 0.001) was more strongly correlated to IR measured by the euglycemic hyperinsulinemic clamp compared to the HOMA1-IR index (r = -0.58, p < 0.002) [8]. In obese children, the efficacy of the HOMA1-IR and HOMA-AD indices in assessing IR, designated as the presence of the metabolic syndrome, was investigated [7]. The AUCs for predicting IR were 0.68 (CI 95%, 0.59–0.76; p < 0.05) and 0.71 (CI 95%, 0.62–0.79; p < 0.05) for HOMA1-IR and HOMA-AD, respectively. All of these AUCs are not susceptible to comparison to the AUCs obtained in the present study due to the large difference in the composition of the samples studied, with respect to age, degree of adiposity and health profile.

In the present study, although stronger coefficients of correlation and higher AUCs were identified for the HOMA-AD index in comparison to the HOMA1-IR, the pairwise comparison between the AUCs showed similar abilities for both of the surrogate indices in identifying IR and metabolic syndrome. Therefore, the addition of the plasma adiponectin levels in the denominator of the HOMA1-IR does not improve the accuracy, and the additional costs may thus not be justified. However, the HOMA-AD may perform differently in other groups that were not studied here such as adolescents, who have an increased IR during puberty, or in severely obese subjects undergoing bariatric surgery whose improvements in IR occurs primarily in the liver and then in the periphery, giving the HOMA-AD index a possible advantage in the detection of this improvement in the long term.

The optimal cutoff identified for the HOMA-AD index for screening IR in women was 0.95. The previous studies that evaluated the HOMA-AD did not investigate the reference values. The cutoffs identified in the screening of IR may be useful in large-scale studies or clinical practice to identify patients with IR.

Nonetheless, our study presents some limitations. There are some reports indicating a differential influence of adiponectin isoforms in insulin sensitivity [19, 20]. High molecular weight adiponectin is the most active form of adiponectin showing better anti-inflammatory, anti-atherogenic, and insulin sensitizing properties [19]. Unfortunately, we did not have information regarding different isoforms in the present study. This is a cross-sectional study, and additional prospective studies will need to verify the HOMA-AD index as a determinant of cardiovascular and diabetes-related events. The sample studied included exclusively adult women. Future studies may investigate the ability of the HOMA-AD index as a surrogate marker of IR in men and even in more specific groups that are stratified according to the metabolic profile. On the other hand, adiponectin is usually greater in women in comparison to men after adjustment for age and total fat mass. On the other hand, this sexual dimorphism has this relative importance reduced after menopause in women probably affecting their overall cardiovascular risk [21]. These information reinforce the validity of our study should be restricted to female population.

Even if HOMA-AD has been validated in children, in hemodialysis patients and in the Japanese population before, this study adds additional information because of its large study population and its multicenter design. Additionally, the present study includes data obtained with the clamp technique, which is the reference method to assess IR.

In conclusion, the HOMA-AD index was demonstrated to be a useful surrogate marker for detecting IR among adult women and presented a similar performance compared with the HOMA1-IR index. These results may assist physicians and researchers in determining which method to use to evaluate IR in light of the available facilities.

## Supporting Information

S1 File BRAMS Data Set. (XLSX)
